# The clinical quandary of left and right ventricular diastolic dysfunction and diastolic heart failure

**DOI:** 10.5830/CVJA-2010-018

**Published:** 2010-08

**Authors:** Ernst R Schwarz, Raja Dashti

**Affiliations:** Cedars Sinai Heart Institute, Cedars-Sinai Medical Center and University of California Los Angeles (UCLA ), Los Angeles, California; Cedars Sinai Heart Institute, Cedars-Sinai Medical Center and University of California Los Angeles (UCLA ), Los Angeles, California

**Keywords:** diastolic heart failure, diastolic dysfunction, congestive heart failure, cardiomyopathy

## Abstract

**Summary:**

Diastolic heart failure is a common clinical entity that is indistinguishable from systolic heart failure without direct evaluation of left ventricular function. Diastolic heart failure is a clinical diagnosis in patients with signs and symptoms of heart failure but with preserved left ventricular function and normal ejection fraction, and is often seen in patients with a long-standing history of hypertension or infiltrative cardiac diseases. In contrast, diastolic dysfunction represents a mechanical malfunction of the relaxation of the left ventricular chamber that is primarily diagnosed by two-dimensional transthoracic echocardiography and usually does not present clinically as heart failure. The abnormal relaxation is usually separated in different degrees, based on the severity of reduction in passive compliance and active myocardial relaxation. The question whether diastolic dysfunction ultimately will lead to diastolic heart failure is critically reviewed, based on data from the literature. Treatment recommendations for diastolic heart failure are primarily targeted at risk reduction and symptom relief. Currently, few data only are reported on diastolic dysfunction and its progression to systolic heart failure.

## Summary

Even though often interchangeably used in the clinical setting, there is a distinction between diastolic dysfunction and diastolic heart failure. A PubMed literature search revealed a total of 1 478 articles using the search terms diastolic heart failure and review. In contrast, only a few randomised controlled trials are available on diastolic heart failure alone. Controversy remains regarding the optimal therapy in patients with either diastolic dysfunction or diastolic heart failure.^1^ An important question is whether diastolic dysfunction does indeed lead to diastolic heart failure and how this progression occurs. Moreover, it is unclear whether diastolic dysfunction results in both diastolic and subsequently, systolic heart failure.

In daily routine, heart failure is often separated into systolic and diastolic failure based on preservation of left ventricular ejection fraction.^1^ The terms ‘heart failure with preserved left ventricular function’ or ‘heart failure with normal ejection fraction’ are utilised to emphasise that the aetiology of the pathophysiology for this group of patients may go beyond diastolic dysfunction alone.^2^

Heart failure in general and diastolic heart failure in particular causes a significant financial burden and increasing consumption of healthcare resources, especially among the elderly population (i.e. for patients 65 years of age or older).^3^,^4^ This article will review the current knowledge of diastolic dysfunction and its progression to diastolic heart failure.

## Diastolic dysfunction

Diastolic dysfunction is a mechanical abnormality brought upon by a breakdown in the passive (compliance) and active (myocardial relaxation) intrinsic properties of the ventricle during diastole. Myocardial hypertrophy (e.g. left ventricular hypertrophy secondary to hypertension) and myocardial ischaemia have been shown to impair the energy-dependant process of myocardial relaxation. The increased afterload in patient with aortic stenosis or hypertension can also inhibit myocardial relaxation by reducing the ability of the left ventricle to contract to small end-systolic volume, and hence limit the ensuing elastic recoil’s ability to enhance myocardial relaxation. Also, diastolic dysfunction can be secondary to pathological states that adversely affect the passive compliance during diastole, such as increases in myocardial wall thickness observed in concentric hypertrophy as a result of longstanding hypertension, or in myocardial fibrosis in patients with infiltrative pathology.^5^

## The role of echocardiography in the assessment of diastolic function

Diastolic function can be evaluated non-invasively using two-dimensional transthoracic echocardiography. The evaluation of left ventricular diastolic function should be an essential part of any echocardiography examination.^6^ The three phases of diastole consist of a period of isovolumic relaxation time (IVRT), followed by an early rapid diastolic filling period (E), a plateau, and finally a late filling due to the atrial contraction or atrial kick (A). These can be evaluated by using the pulse wave (PW) Doppler of the mitral valve and pulmonary veins. The left ventricular filling pattern obtained will therefore indirectly reflect the left ventricular filling pressures.

**Fig. 1.**
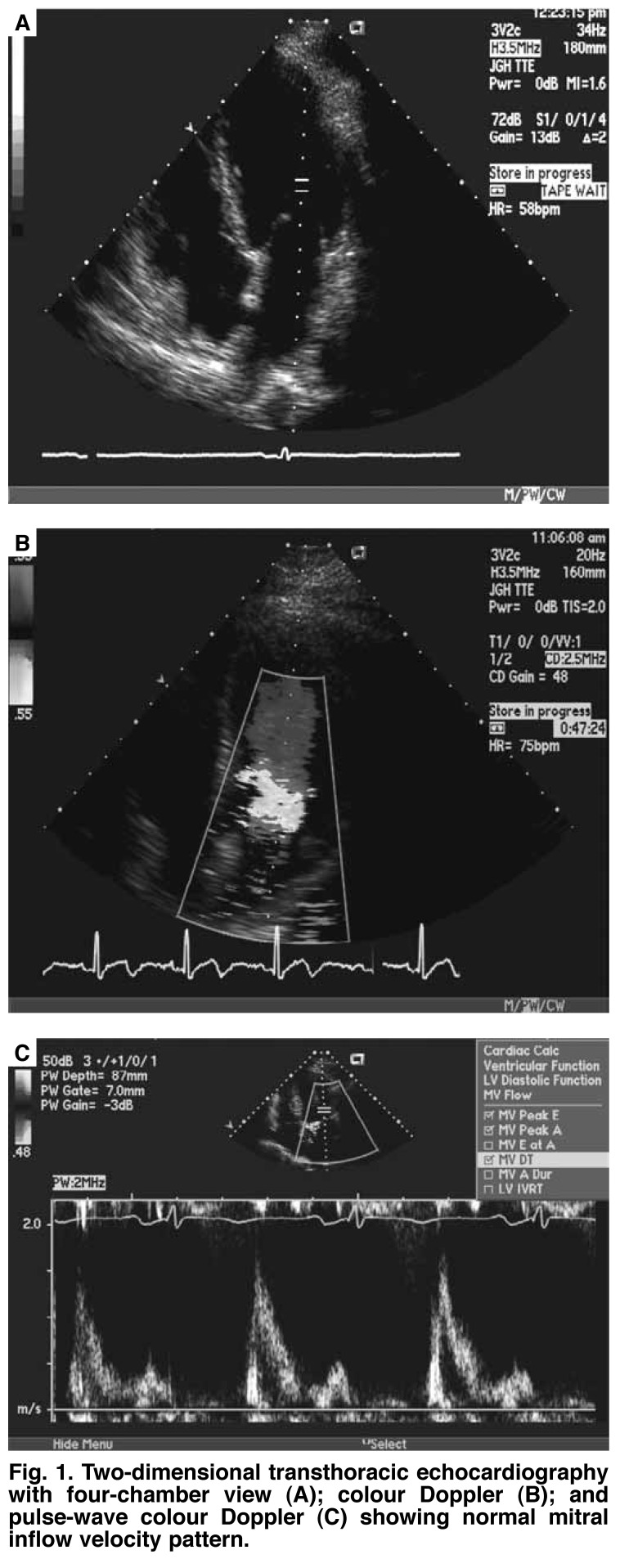
Two-dimensional transthoracic echocardiography with four-chamber view (A); colour Doppler (B); and pulse-wave colour Doppler (C) showing normal mitral inflow velocity pattern.

**Fig. 2.**
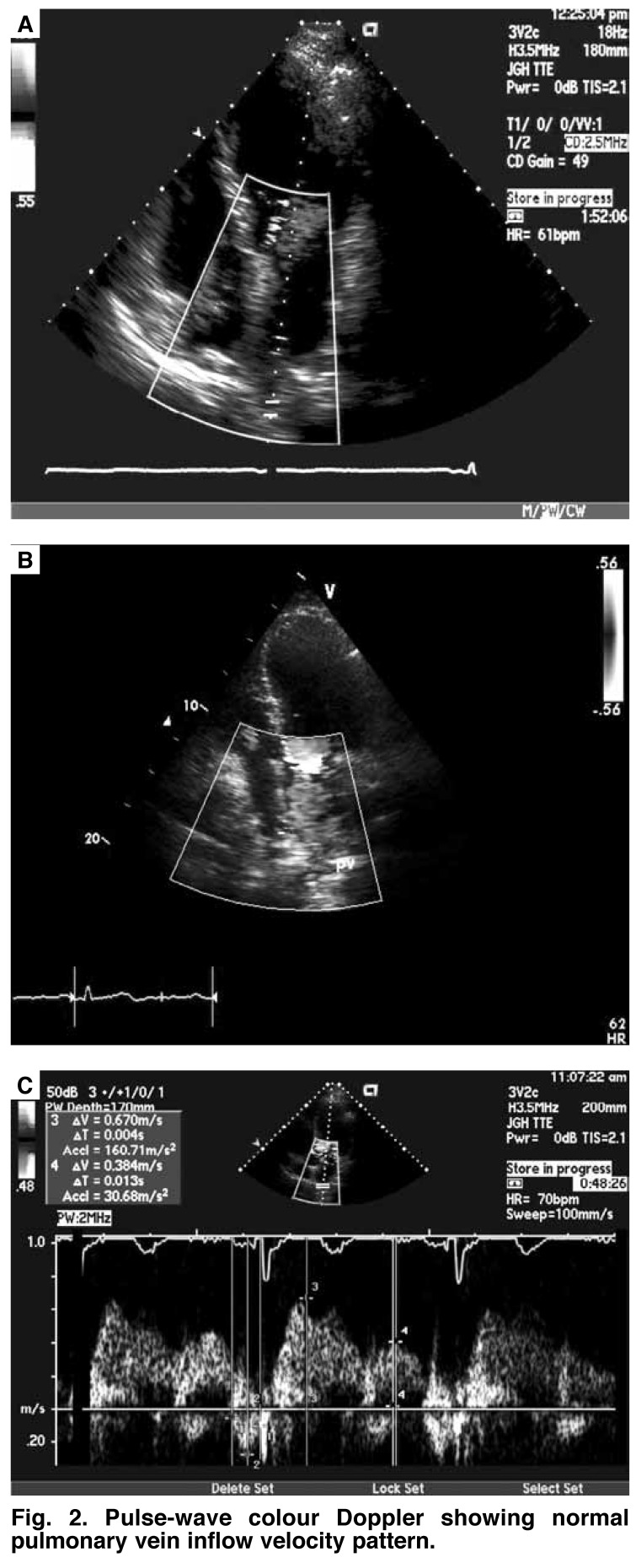
Pulse-wave colour Doppler showing normal pulmonary vein inflow velocity pattern.

A complete left ventricular diastolic assessment should include assessment of the IVRT, peak E velocity, peak A velocity, E/A ratio, deceleration time (DT), and A duration, which are obtained from the transmitral inflow velocities (Fig. 1). Pulmonary vein (PV) flow velocities are then measured, which include four components: two systolic velocities (PVs1 and PVs2), diastolic velocity (PVd), and atrial flow reversal (PVa) (Fig. 2).^7^

**Fig. 3.**
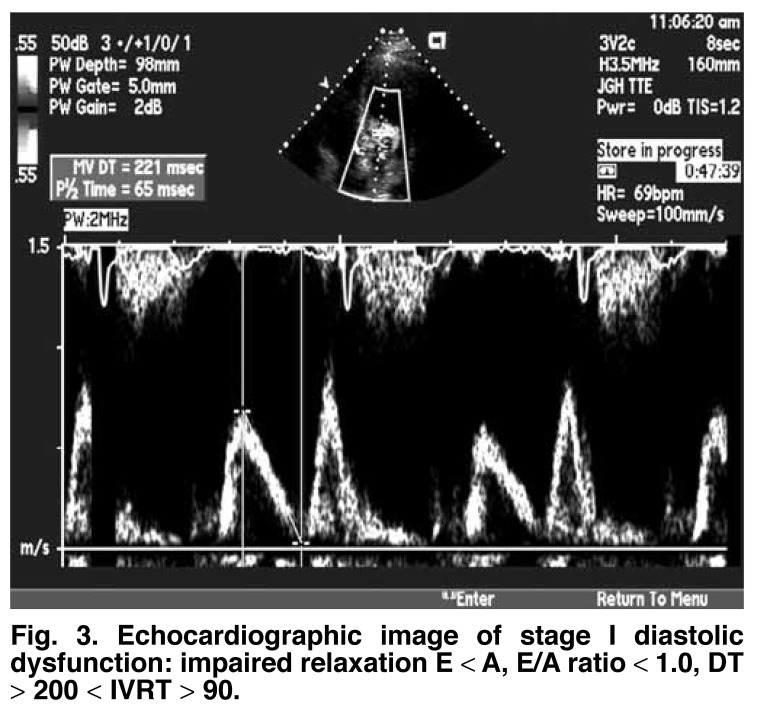
Echocardiographic image of stage I diastolic dysfunction: impaired relaxation E < A, E/A ratio < 1.0, DT > 200 < IVRT > 90.

**Fig. 4.**
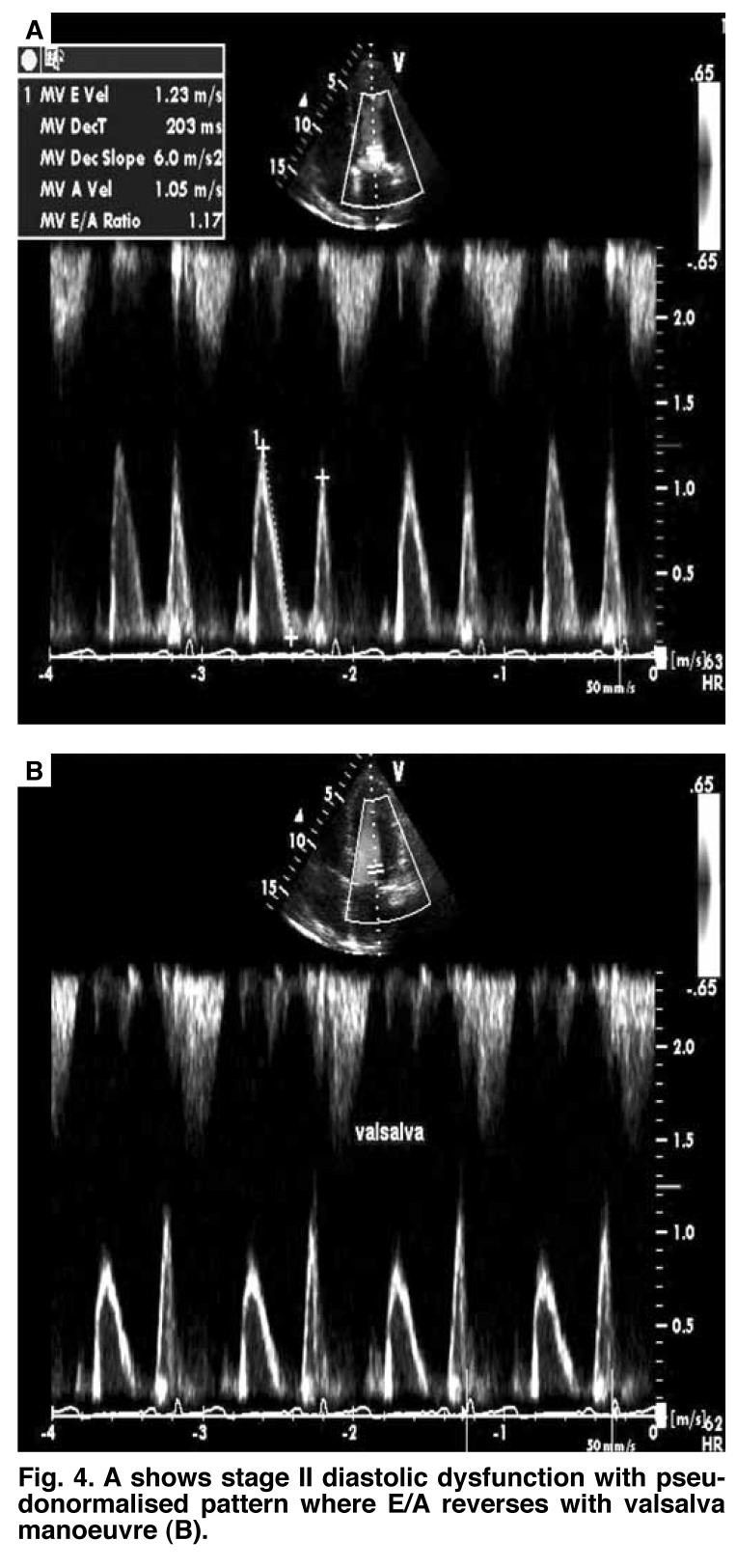
A shows stage II diastolic dysfunction with pseudonormalised pattern where E/A reverses with valsalva manoeuvre (B).

**Fig. 5.**
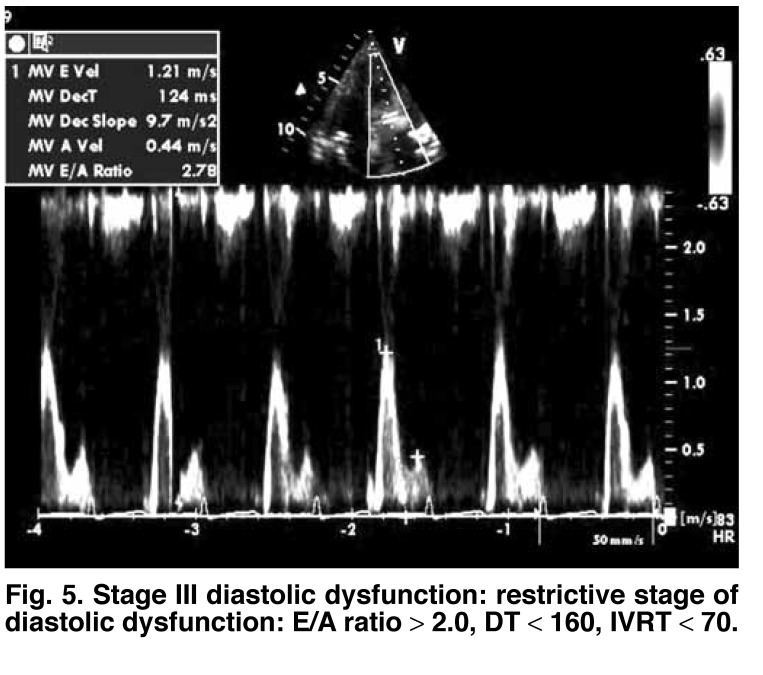
Stage III diastolic dysfunction: restrictive stage of diastolic dysfunction: E/A ratio > 2.0, DT < 160, IVRT < 70.

**Fig. 6.**
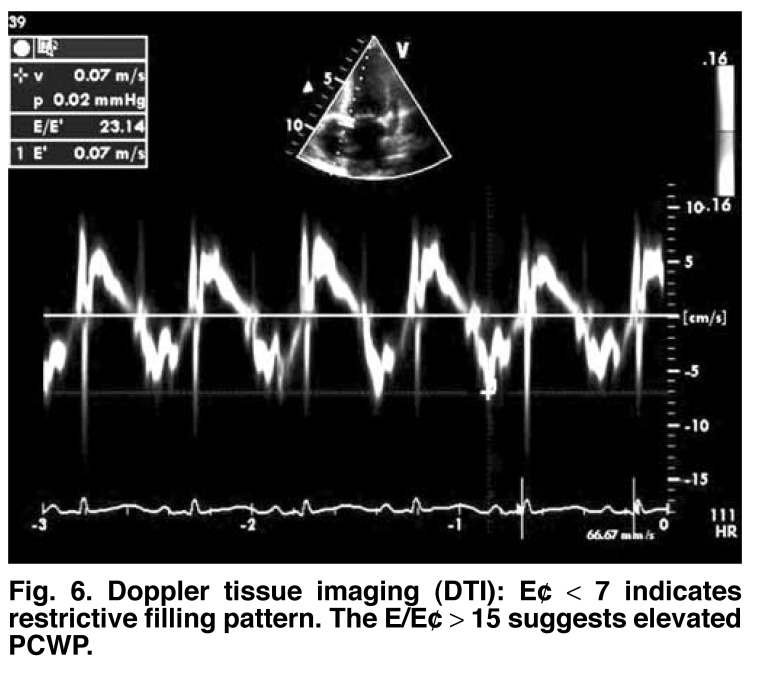
Doppler tissue imaging (DTI ): E¢ < 7 indicates restrictive filling pattern. The E/E¢ > 15 suggests elevated PCWP.

Based on the echocardiographic parameters, diastolic dysfunction has been divided into three different grades of severity of ventricular compliance, relaxation rate and filling pressures.^8^ Stage one is the mildest form of diastolic dysfunction with delayed relaxation defined by an early filling to late or atrial filling (E/A) ratio less than 1, prolonged IVRT and prolonged DT. The systolic to diastolic pulmonary venous (S/D) ratio is greater than 1 (Fig. 3). Stage two is marked by a moderate level of dysfunction and defined by E/A of greater than 1 and/or greater than 2 with S/D less than 1, and is often called pseudonormalisation (with a normal diastolic filling pattern), caused by elevated left atrial pressures. This can be unmasked by reducing preload, for example by use of the Valsalva manoeuvre or application of sublingual nitroglycerine (Fig. 4). Stage three is marked by a restrictive filling pattern and signifies severe diastolic dysfunction, i.e. decreased compliance and marked increase in left atrial pressure. The E/A is greater than 2, IVRT and DT are short, and S/D is less than 1 (Fig. 5). The mitral A duration is shorter than the PVa duration (Fig. 6).

**Fig. 7.**
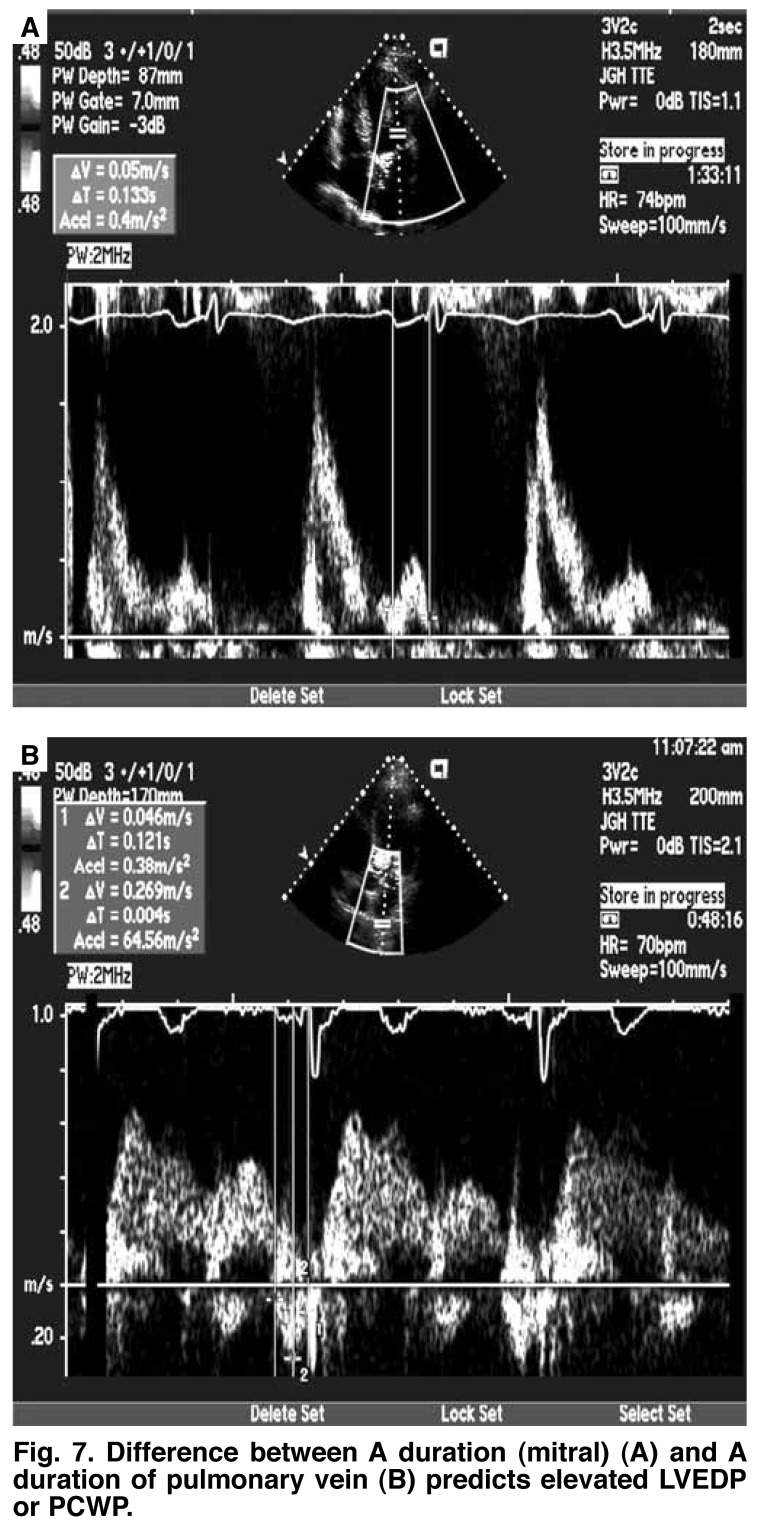
Difference between A duration (mitral) (A) and A duration of pulmonary vein (B) predicts elevated LV EDP or PCWP.

Mitral annular velocity by tissue Doppler imaging also has been used to assess diastolic function. This is referred to as E. The Em (mitral)/E¢ (annular) ratio has been found to correlate well with increased pulmonary capillary wedge pressure (PCWP). The E/E¢ ratio is normally less than 8. The E¢ is shown to be low, in restrictive stage less than 8. A ratio of greater than 15 indicates elevated PCWP (Fig. 7).^9^

Although rarely performed for evaluation of diastolic dysfunction alone, the most accurate invasive diagnostic technique is cardiac catheterisation with direct measurements of left ventricular end-diastolic pressure.^10^,^11^ Parameters of chamber stiffness are correlated with changes in pressure to changes in chamber volume.

## Left ventricular diastolic dysfunction

In its simplest form, left ventricular diastolic dysfunction is defined as impairment in the capacity of the left ventricle to accept blood without a compensatory increase in left atrial pressure.^12^ Patients with left ventricular diastolic dysfunction tend to have elevated left ventricular diastolic pressure in the presence of normal or even reduced left ventricular volume, as the pressure–volume curve in these patients is shifted upwards.^13^,^14^ Over the years, a variety of co-morbid conditions have been associated with development of left ventricular diastolic dysfunction, such as myocardial scarring, transmural myocardial infarction, chronic constrictive pericarditis, chronic coronary artery disease, dilated cardiomyopathy, hypertrophic cardiomyopathy, diabetic cardiomyopathy, hypertension, aortic stenosis as well as normal aging.^12^

The underlying connection in the possible aetiologies of left ventricular diastolic dysfunction is their ability to hinder one or both of the intrinsic diastolic properties of compliance or relaxation. Pathological states such as fibrosis and concentric hypertrophy can reduce compliance of the myocardium by increasing passive ventricular stiffness, thereby affecting the passive property of compliance in diastole. Ischaemia and disease processes leading to increased afterload affect diastole by impairment of the active rate of relaxation.

## Left ventricular diastolic dysfunction and heart failure

The prevalence as well as overall significance of diastolic heart failure has become distinctly apparent. Diastolic heart failure was originally reported in 1937 when Fishberg referred to it as ‘hypodiastolic failure’, a form of cardiac insufficiency secondary to inadequate filling of the left ventricle during diastole.^15^ A half a century later, Kessler became the first to discuss the clinical syndrome of diastolic heart failure.^16^ Over the years, a number of landmark publications have guided our current understanding in diagnosing diastolic heart failure.

Recognising the difficulty of non-invasive assessment of the LV diastolic function, in 2000, Vasan and Levy proposed a classification scheme for diagnosis of diastolic heart failure in the hope of reducing the difficulty of diagnosis of this rather prevalent pathology.^17^ According to the degree of diagnostic certainty, patients were partitioned into possible, probable, or definite diastolic heart failure. While keeping the need for evidence of heart failure for all categories, the diagnosis of probable or definite diastolic heart failure required evidence of normal left ventricular systolic function within three days of the initial heart failure event. Most importantly it was argued that ‘evidence of abnormal LV relaxation, filling, diastolic distensibility, or diastolic stiffness’ is required for a definite diagnosis of diastolic heart failure.

More recently, Zile and colleagues have published several prospective studies, concluding that the diagnosis of diastolic heart failure does not require objective recording of left ventricular diastolic dysfunction but only documentation of preserved systolic function. In two separate studies utilising both Doppler echocardiography and cardiac catheterisation, the authors observed a statistically significant percentage of patients with clinical diagnosis of heart failure and normal ejection fraction (EF > 45%) to be suffering from abnormalities in active relaxation or passive compliance.^18^,^19^

The degree of involvement that left ventricular diastolic dysfunction plays in preserved ejection fraction heart failure is debatable and has been the major argument made by those that believe diastolic heart failure is the correct diagnosis for patients with heart failure and normal ejection fraction, given that these patients do not suffer from significant valvular, pericardial or pulmonary disease. Left ventricular diastolic dysfunction has also been found to be present in patients with heart failure and reduced ejection fraction, a form of heart failure that was originally believed to be mainly secondary to a systolic dysfunction pathophysiology.^20^

## Clinical studies in patients with diastolic dysfunction

In 1972 Gaasch and colleagues performed some of the first studies to evaluate the possible effects of left ventricular diastolic dysfunction. The authors described that left ventricular diastolic dysfunction has a negative impact on systolic function through its limitation of the Frank-Starling mechanism.^21^ Patients with conditions such as left ventricular hypertrophy have elevated left ventricular end-diastolic pressure and decreased compliance, which affects the length–tension relationship by decreasing muscle fibre stretch at any given peak systolic stress. This might explain why decreased exercise tolerance is one of the first clinical symptoms associated with echocardiographically diagnosed diastolic dysfunction.

Exercise tolerance in patients with left ventricular diastolic dysfunction who are asymptomatic at rest may be compromised secondary to the inability to enhance diastolic filling by the degree necessary to increase the cardiac output during exercise without causing an abnormal elevation in left atrial pressure. Diastolic dysfunction has been found to be aggravated by exercise, especially with an increase in blood pressure. Recent studies have observed the development of left ventricular diastolic dysfunction in the presence of hypertension prior to the development of ventricular hypertrophy.^22^,^23^ Left ventricular diastolic dysfunction can therefore represent myocardial end-organ damage prior to progression to clinically relevant heart failure, although further trials are needed to support this hypothesis.

The magnitude of asymptomatic left ventricular diastolic dysfunction in the general population is still unclear. In an attempt to determine the prevalence of pre-clinical diastolic dysfunction, Redfield *et al*. performed a cross-sectional survey of 2 042 randomly selected residents over the age of 45 years in Olmsted County, Minnesota.^24^ The authors found the prevalence of asymptomatic echocardiographically diagnosed diastolic dysfunction to be 28%, with an increased prevalence seen in older patients, diabetics, and in patients with cardiovascular disease (hypertension, coronary artery disease, cardiomyopathies).

A prospective trial in 206 patients with the clinical diagnosis of heart failure (New York Heart Association Grade II or higher) reported that, based on echocardiographic parameters, 91% of 102 patients with an EF greater than 50% had some degree of diastolic dysfunction, and 92% of 71 patients with an EF of less than 40% suffered from left ventricular diastolic dysfunction.^25^ Patients with reduced ejection fraction were more likely to have moderate to severe diastolic dysfunction in comparison to patients with preserved ejection fraction (27 vs 62%, respectively).

In patients with heart failure with preserved EF, left ventricular diastolic dysfunction was accompanied by left ventricular hypertrophy, while in patients with heart failure and reduced EF, left ventricular diastolic dysfunction was associated with left ventricular dilation and marked systolic dysfunction. The overall prognosis and mortality appears to be significantly influenced by the degree of left ventricular diastolic dysfunction in heart failure patients, regardless of ejection fraction.^26^

## Clinical studies in patients with diastolic heart failure

The American College of Cardiology and the American Heart Association (ACC/AHA) Task Force has previously stated that a definitive diagnosis can be made in heart failure patients with preserved EF if there is a decreased rate of ventricular relaxation with elevated LV filling pressure, clarifying the need for coexistence of normal contractility (LV systolic function) and LV volume.^27^ In further assessing such assumptions, Baicu *et al.* compared 75 patients with heart failure and normal ejection fraction with 75 patients without cardiovascular disease.

After analysing both echocardiographic parameters and data derived from cardiac catheterisation, it appeared that left ventricular systolic function, contractility and performance was intact in patients with presumed diastolic heart failure (with normal ejection fraction).^28^ In a review of data on left ventricular structure and function in heart failure patients with normal ejection fraction and hypertension, Zile and Lewinter have argued that left ventricular end-diastolic volume is within the normal range in patients with diastolic heart failure.^29^

The frequently quoted CHARM-preserved trial was one of two large trials, studying a total of 3 023 patients with heart failure with preserved EF of more than 40%, treated with the angiotensin receptor blocker candesartan.^30^ After a median follow up of 36.6 months, fewer candesartan-treated patients were hospitalised for heart failure compared with the placebo group (402 vs 566, *p* = 0.014), but there was no significant difference with regard to cardiovascular mortality. As an important finding on the side, a significant 40% reduction was seen in the development of new diabetes mellitus in the candesartan group compared with the placebo group (4 vs 7%, *p* = 0.005). This has gained even more interest in view of recently published data on diabetes as an independent predictor of cardiovascular morbidity and mortality in heart failure patients, regardless of their EF,^31^ underlining the importance of controlling risks and co-morbidities in patients with diastolic heart failure.

The I-PRESERVE trial, published in 2008, studied 4 128 heart failure patients 60 years of age and older with an EF of at least 45% who were randomly assigned to receive 300 mg of the angiotensin receptor antagonist irbesartan or placebo. Primary event rates as assessed as a composite of death from any cause or hospitalisation for a cardiovascular cause in the irbesartan and placebo groups were 100.4 and 105.4 per 1 000 patient-years, respectively,^32^ which was not significantly different. In conclusion, neither candesartan (in the CHARM preserved trial) nor irbesartan (in I-PRESERVE) improved survival in these large trials in patients with pure diastolic heart failure.

## Right ventricular diastolic dysfunction

Similar to left ventricular diastolic dysfunction, there have been multiple aetiologies associated with impairment in mechanical compliance as well as relaxation parameters that lead to right ventricular diastolic dysfunction. Over the years, right ventricular diastolic dysfunction has been observed in a variety of settings, including obesity, cystic fibrosis, chronic aortic stenosis, arterial hypertension and Chagas disease.^33^-^36^ Studies investigating the functional parameters of the right ventricle during diastole were slow to formulate due to the difficulty of correctly measuring right ventricular volume prior to the advent of Doppler echocardiography.^37^ The algorithm used for assessment and diagnosis of right ventricular diastolic dysfunction with Doppler echocardiography utilises pulsed-wave Doppler of the transtricuspid flow, hepatic venous flow and tissue Doppler imaging of the tricuspid annulus or tricuspid annular velocity.^38^

Normal hepatic venous flow is defined as a ratio of systolic to diastolic velocities greater than one with the atrial wave reversal less than half the maximum systolic wave velocity.^39^ Mild right ventricular diastolic dysfunction is defined by E/A < 1 in transtricuspid flow velocities, or 1 < E/A < 2 with S/D > 1 in hepatic vein flow and early component of the tricuspid annular tissue Doppler velocity (Et) less than atrial component of the tricuspid annular tissue Doppler velocity (At), or an atrial reversal wave more than half of the systolic wave of the hepatic vein flow.

Moderate or severe right ventricular diastolic dysfunction can be assumed to be present if a reduced or inverted systolic waveform, respectively, is present on the Doppler hepatic vein flow signal. Studies on pulmonary hypertension patients have led to the speculation that right ventricular diastolic dysfunction may be an independent factor contributing to right heart failure and death in patients with pulmonary hypertension.^40^

Gan *et al.* showed that in patients with pulmonary hypertension, the increase in right ventricular afterload resulted in ventricular hypertrophy and right ventricular diastolic dysfunction.^41^ The degree of diastolic dysfunction correlated with the severity of pulmonary hypertension, which improved with medical therapy that reduced afterload.

Right ventricular diastolic dysfunction in the setting of heart failure was first reported by Riggs in 1993.^42^ The author reported impaired right ventricular filling parameters in six children with dilated cardiomyopathy. Yu *et al.* published the first study that systematically assessed right ventricular diastolic dysfunction in 1996; comparing 114 patients with symptomatic heart failure (EF < 50%) with 31 patients with pulmonary hypertension (pulmonary systolic artery pressure > 40 mmHg) as well as 40 healthy subjects.^43^ The authors described a significant number of patients with systolic heart failure and/or pulmonary hypertension suffering from right ventricular diastolic dysfunction. Even after exclusion of patients with pulmonary hypertension, a statistically significant percentage of heart failure patients suffered right ventricular diastolic dysfunction.

In their analysis of 105 patients with systolic heart failure, Yu and Sanderson demonstrated right ventricular diastolic dysfunction to be present in 21% of patients as assessed by echocardiography.^44^ Although a low-powered study, the authors concluded that right ventricular diastolic dysfunction was an independent predictor for non-fatal hospital admissions for unstable angina or heart failure, even though it was not observed to be a prognostic factor for mortality, either alone or in combination with left ventricular diastolic dysfunction.

## Right and left ventricular interaction in diastolic dysfunction

The French physician Bernheim was one of the first to report the concept of ventricular interdependence in 1910, noting that right ventricular performance can be compromised through compression of the right ventricle by a dilated or hypertrophied left ventricle.^46^ In 1956, Dexter explained a possible mechanism for diastolic interdependence.^46^ The ‘reverse Bernheim effect’ hypothesised an increase in right ventricular volume secondary to an atrial septal defect, which can cause the septum to be displaced toward the left ventricular cavity and inhibit left ventricular filling mechanisms. A decade later in 1967, Taylor *et al.* reported that the distension of one ventricle during diastole can affect the compliance of the neighbouring ventricle.^47^

The term diastolic ventricular interaction refers to the concept that compliance of one ventricle is influenced through a shared septum by the changes in volume, pressure, and/or compliance of the other ventricle.^47^ Although there are implications that diastolic ventricular interaction plays a role in exercise intolerance in patients with systolic heart failure, we currently do not have a great understanding of the possible role it may have in patients with diastolic heart failure. Ventricular interactions have been reported indirectly in patients with pathology of one ventricle and diastolic dysfunction of the neighbouring ventricle.

Right ventricular diastolic dysfunction has been observed in pathological conditions that result in elevated left ventricular pressure, such as systemic hypertension, aortic stenosis and hypertrophic cardiomyopathy.^38^,^48^,^49^ The reverse has also been reported in patients with elevated right ventricular volume or pressure with impaired left ventricular diastolic function.^50^ Furthermore, it has been suggested that right ventricular diastolic dysfunction observed in patients with heart failure but normal pulmonary artery pressures may be caused indirectly by coexistent left ventricular diastolic dysfunction secondary to ventricular interdependence.^51^ Although a realistic prospect, the possible role that diastolic ventricular interaction may play in the potential progression from diastolic dysfunction to clinical heart failure is currently not well established.

## Progression of diastolic heart failure

In 2001, Aurigemma *et al.* published the possible rate of progression from asymptomatic diastolic dysfunction to clinical heart failure.^52^ The study analysed 2 671 individuals without coronary heart disease, congestive heart failure or atrial fibrillation. At baseline, 15% of the patients had diastolic dysfunction, determined by echocardiography, with 170 participants eventually developing heart failure after a five-year follow-up period (6.4%), concluding that echocardiographic findings can be suggestive of the development of heart failure.

Despite arguments regarding exercise limitations and left ventricular diastolic dysfunction representing a possible early marker of myocardial damage, the rate of progression from diastolic dysfunction to diastolic heart failure remains uncertain. Currently there are no large clinical trials assessing the possible progression from asymptomatic right ventricular diastolic dysfunction to clinical right ventricular failure.

## Guidelines and therapy for diastolic heart failure

The difficulties in the diagnosis of diastolic heart failure have been partly responsible for the limited number of larger randomised, controlled trials to guide treatment. In 1998, the European study group published one of the first widely analysed guidelines for diagnosis of diastolic heart failure, stating the need for evidence of heart failure with normal systolic function (LVEF ≥ 0.50) as well as evidence of abnormal filling, diastolic distensibility, LV relaxation or diastolic stiffness.^53^

The European Society of Cardiology recently published their latest guidelines for diagnosis of diastolic heart failure in 2007; providing specific guidelines on how to diagnose and exclude heart failure with normal ejection fraction.^54^ The guidelines have three major criteria for diagnosing heart failure with normal ejection fraction; (1) signs/symptoms of heart failure, (2) normal or mildly reduced systolic function (EF > 50% with a left ventricular end-diastolic volume index less than 97 ml/m^2^) and (3) evidence of left ventricular diastolic dysfunction.

The diagnostic strategy provided in this set of guidelines allows for non-invasive methods of assessing for left ventricular diastolic dysfunction through tissue Doppler parameters (early mitral valve flow velocity to early tissue Doppler lengthening velocity (E/E¢ > 15) and routine blood test biomarkers (brain natriuretic peptide > 200 pg/ml) to play a role in situations when invasive haemodynamic measurements (LV end-diastolic pressure > 16 mmHg or mean pulmonary capillary wedge pressure > 12 mmHg) are not available.

Current treatment of diastolic heart failure has been aimed at controlling blood pressure and tachycardia, using diuretics to control pulmonary congestion and peripheral oedema, and alleviation of myocardial ischaemia. The ACC/AHA also recommend using beta-adrenergic blocking agents, angiotensin receptor blockers, angiotensin converting enzyme inhibitors, calcium antagonists in those patients with controlled blood pressure, and digitalis in order to control heart failure symptoms. In the latest update of the ACC/AHA practice guidelines for the diagnosis and management of chronic heart failure in the adult, which comprises a document of 63 pages, the treatment of diastolic heart failure is summarised in less than one page.^55^

Chinnaiyan *et al.* described the combined use of beta-blockers, angiotensin converting enzyme inhibitors, angiotensin II receptor blockers, calcium channel blockers and spironolactone as potential disease-modifying therapy.^56^ The authors believe that the effects of these drugs improve diastolic dysfunction and diastolic heart failure by regression of left ventricular hypertrophy and decreased collagen content. They recommend these drugs to be utilised in both the setting of decompensated diastolic heart failure as well as for the chronic outpatient management of diastolic heart failure. In the recently published Hong Kong diastolic heart failure study, 150 patients with heart failure and preserved ejection fraction were randomised to diuretics, ACE inhibitors or angiotensin II receptor blocker therapy.^57^ Only diuretic therapy reduced symptoms and improved quality of life during one-year follow up.

Currently, only a few large randomised clinical trials have assessed the possible benefit of pharmacotherapy at different stages of non-invasively diagnosed diastolic dysfunction, such as the CHARM Preserved trial and I-PRESERVE (see above).^30^,^32^ While hospitalisation rates have been reduced with candesartan therapy, survival rate mortality has not been improved in either of these trials.

Some small trials have been carried out in an attempt to evaluate possible benefits of pharmacotherapy for patients with left ventricular diastolic dysfunction and decreased exercise tolerance. Warner *et al*. studied 20 patients with mild diastolic dysfunction, diagnosed by Doppler echocardiography, with a marked hypertensive response to exercise.^58^ The authors reported that using the angiotensin II receptor blocker losartan, resting blood pressure was unchanged but the hypertensive response to exercise was reduced (from a mean systolic blood pressure (SBP) of 226 mmHg to a mean SBP of 193 mmHg).

Similar studies confirmed the benefits of angiotensin II receptor blockers on exercise tolerance by comparing its effects with calcium channel blockers (verapamil) or diuretics (hydrochlorothiazide). In two separate trials, Little *et al.* demonstrated that angiotensin II receptor blockers, calcium channel blockers and diuretics all have the ability to blunt an increase in SBP during exercise in patients with asymptomatic left ventricular diastolic dysfunction, but only angiotensin II receptor blocker therapy increased exercise duration and improved quality of life, as assessed by questionnaires.^59^,^60^

## Conclusion

Further research is needed to improve current knowledge of diastolic dysfunction and diastolic heart failure as well as its progression over time. The management of diastolic heart failure is currently aimed at symptomatic management and control of physiological factors known to affect ventricular relaxation, and control of risk factors and co-morbidities (such as hypertension and diabetes mellitus). A timeline for initiation of treatment for diastolic dysfunction has yet to be defined. It is anticipated but not proven whether early initiation of pharmacotherapy once diastolic dysfunction has been diagnosed even in the absence of symptoms will prevent or delay the onset of symptomatic heart failure.
